# A pragmatic multi-centre randomised controlled trial of fluid loading in high-risk surgical patients undergoing major elective surgery - the FOCCUS study

**DOI:** 10.1186/cc10592

**Published:** 2011-12-16

**Authors:** Brian H Cuthbertson, Marion K Campbell, Stephen A Stott, Andrew Elders, Rodolfo Hernández, Dwayne Boyers, John Norrie, John Kinsella, Julie Brittenden, Jonathan Cook, Daniela Rae, Seonaidh C Cotton, David Alcorn, Jennifer Addison, Adrian Grant

**Affiliations:** 1 Department of Critical Care Medicine, Sunnybrook Health Sciences Centre, 2075 Bayview Avenue, Room D132, Toronto, ON M4N 3M5, Canada; 2 Health Services Research Unit, Health Sciences Building, University of Aberdeen, Ashgrove Road, Foresterhill, Aberdeen, AB25 2ZD, UK; 3 Intensive Care Unit, Aberdeen Royal Infirmary, Westburn Road, Aberdeen, AB25 2ZN, UK; 4 Health Economics Research Unit, Polwarth Building, University of Aberdeen, Foresterhill, Aberdeen, AB25 2ZD, UK; 5 Section of Anaesthesia Pain and Critical Care, University of Glasgow, 4th Floor, Walton Building, Glasgow Royal Infirmary, 91 Wishart Street, Glasgow, G31 2HT, UK; 6 Department of Surgery, University of Aberdeen, Polwarth Building, Foresterhill Aberdeen, AB25 2ZD, UK; 7 Royal Alexandra Hospital, Corsebar Road, Paisley, Strathclyde, PA2 9PN, UK; 8 Institute of Applied Health Sciences, Health Sciences Building, University of Aberdeen, King's College, Foresterhill, Aberdeen, AB24 3FX, UK

**Keywords:** surgery, peri-operative management, fluid therapy, outcome, morbidity, optimisation

## Abstract

**Introduction:**

Fluid strategies may impact on patient outcomes in major elective surgery. We aimed to study the effectiveness and cost-effectiveness of pre-operative fluid loading in high-risk surgical patients undergoing major elective surgery.

**Methods:**

This was a pragmatic, non-blinded, multi-centre, randomised, controlled trial. We sought to recruit 128 consecutive high-risk surgical patients undergoing major abdominal surgery. The patients underwent pre-operative fluid loading with 25 ml/kg of Ringer's solution in the six hours before surgery. The control group had no pre-operative fluid loading. The primary outcome was the number of hospital days after surgery with cost-effectiveness as a secondary outcome.

**Results:**

A total of 111 patients were recruited within the study time frame in agreement with the funder. The median pre-operative fluid loading volume was 1,875 ml (IQR 1,375 to 2,025) in the fluid group compared to 0 (IQR 0 to 0) in controls with days in hospital after surgery 12.2 (SD 11.5) days compared to 17.4 (SD 20.0) and an adjusted mean difference of 5.5 days (median 2.2 days; 95% CI -0.44 to 11.44; *P *= 0.07). There was a reduction in adverse events in the fluid intervention group (*P *= 0.048) and no increase in fluid based complications. The intervention was less costly and more effective (adjusted average cost saving: £2,047; adjusted average gain in benefit: 0.0431 quality adjusted life year (QALY)) and has a high probability of being cost-effective.

**Conclusions:**

Pre-operative intravenous fluid loading leads to a non-significant reduction in hospital length of stay after high-risk major surgery and is likely to be cost-effective. Confirmatory work is required to determine whether these effects are reproducible, and to confirm whether this simple intervention could allow more cost-effective delivery of care.

**Trial registration:**

Prospective Clinical Trials, ISRCTN32188676

## Introduction

High-risk patients undergoing major surgery are at significant risk of death or major morbidity [1,2]. One of the largest bodies of evidence in this field is around "pre-operative optimisation" in major high risk surgery [3-5]. This label characterises a highly complex intervention which comprises a raft of intervention components. These include: pre- and post-operative admission to an ICU; pre-operative placement and monitoring with a pulmonary artery catheter; pre-operative intravenous fluid loading followed by inotropic support to achieve and maintain supranormal cardiac indices and oxygen delivery targets [3-7]. Randomised studies have suggested a significant outcome advantage from this strategy [3-6]. In this group, meta-analysis of randomised controlled trials suggests that pre-operative optimisation improves morbidity and mortality as well as reduces hospital length of stay [7].

However, despite this evidence base, pre-operative optimisation has failed to have significant penetration into clinical practice with apparent low levels of implementation in most countries. Reasons for this failure to implement are unclear but lack of ICU beds for patients prior to surgery may be a major factor in some countries [8]. This inability to implement this intervention may, at least in part, have led to a move towards peri-operative and post-operative optimisation strategies [9-14].

These strategies also aim to target goals for cardiac index and oxygen delivery using a variety of fluid and inotropic interventions targeted via a range of cardiac output monitoring devices [9-14]. There is evidence that a variety of these optimisation strategies may be of benefit in terms of hospital length of stay but there is a lack of evidence for important improvements in mortality or on the cost-effectiveness of care. A further reason clinical teams may not have implemented these strategies includes the growing data on fluid restrictive strategies in major surgery [15-18]. This literature supplies evidence that peri-operative restriction of fluid may improve outcome from major surgery. This evidence, at least superficially, seems contradictory to the pre-operative optimisation evidence and this has split opinions on the optimal peri-operative fluid management strategy [19].

It is unclear from this evidence base whether the benefit from these interventions can be attributed to the entire intervention package or whether the benefits can be obtained from individual or combined components of these interventions. We hypothesised that fluid loading would be associated with benefit in high-risk surgical patients. We aimed to test whether ward-based pre-operative fluid loading cost-effectively shortened hospital length of stay in high-risk surgical patients undergoing major elective surgery.

## Materials and methods

This was a multi-centre, prospective, randomised, controlled trial conducted in four Scottish hospitals, coordinated from the Centre for Healthcare Randomised Trials in the Health Services Research Unit, University of Aberdeen [20]. The trial was designed as a partial 2*2 factorial design with initial randomisation to fluid loading versus no fluid loading, with a secondary randomisation (if an ICU bed was available at the time of recruitment) to intensive care (level 3) versus high dependency (level 2) care [20]. The second randomisation had to be abandoned, however, due to a continuing lack of ICU beds at the time of patient recruitment (this decision was agreed upon with the funder, the Data Monitoring Committee (DMC) and the Trial Safety Committee (TSC). As such, this paper presents the results for the single comparison of fluid loading versus no fluid loading. The design features and estimates used in this protocol were informed by the results of a 15-week pilot program undertaken in the Aberdeen Royal Infirmary during which 23 patients were recruited (not included in this analysis). The main study was undertaken in three university hospitals (Aberdeen Royal Infirmary, Glasgow Royal Infirmary and Glasgow Western Infirmary) and one district general hospital (Royal Alexandra Hospital, Paisley).

Participants were recruited from the elective operating schedules in the recruiting hospitals and informed consent was obtained. Patients were, in general, identified following a pre-assessment visit at a local hospital or following their initial appointment with the surgeon. They were sent an information leaflet prior to their hospital admission whenever possible. Informed consent was sought from these patients on their admission to hospital. Patients were fasted according to local hospital fasting policies and always for at least six hours before surgery. No hospital in the study allowed consumption of carbohydrate rich fluids up to two hours before surgery.

Inclusion criteria included patients undergoing major elective intra-abdominal surgery including major intra-peritoneal surgery, major open aortic surgery, major renal and bladder surgery and hysterectomy and oophorectomy for cancer. Patients were required to fulfil two high-risk surgical criteria according to the Revised Cardiac Risk Index (RCRI); these include a high risk type of surgery, presence of ischaemic heart disease, history of congestive heart failure, history of cerebrovascular disease, insulin therapy for diabetes and pre-operative serum creatinine > 160 μmol/L [21]. Patients who underwent open or laparoscopically-assisted surgery were eligible. Clinical exclusion criteria included clinician concern about the safety of interventions, New York Heart Association grade IV heart failure, emergency surgery, chronic renal failure/creatinine > 300 μmol/L, lack of informed consent, age < 16 years, pregnancy, major hepatic surgery, and expected survival < 6 months.

Participants were randomised through an interactive voice response automated telephone randomisation service on the day before surgery. A minimisation algorithm was used, incorporating centre, age, sex and type of surgery [22]. POSSUM (Pre-Operative and Operative Physiological and Operative Severity Score for Enumeration of Mortality and Morbidity) scores were measured to derive physiological disturbance and operative severity [23].

### Fluid interventions

Patients were randomised to ward-based intravenous pre-operative fluid loading or no pre-operative fluid loading. The pre-operative fluid loading group patients were electively commenced on 25 ml/kg Ringer's lactate solution over the six-hour period in the ward setting before surgery [5]. In the standard fluid regimen no routine pre-operative fluid loading was given. All patients receiving bowel preparation were given 10 ml/kg Ringer's lactate solution in the 12- to 6-hour period before surgery irrespective of trial group allocation as this is deemed to be the best clinical practice. Fluids were not warmed during administration. This meant that fluid loading patients who received bowel preparation would receive 35 ml/kg in the 12 hours before surgery.

All non-protocol fluid prescriptions and other management decisions were made by the clinically responsible surgical team. We did not control or protocolise the time of discharge from hospital. Due to the nature of the interventions no blinding of the interventions was possible.

All participants were followed up daily for one week for major morbidity and mortality, then at hospital discharge and then one, three and six months after surgery for survival and quality of life. The decision to discharge the patient from hospital was made by the caring team with no involvement of the study personnel. Study outcomes measured during hospital stay were measured by study personnel not blinded to the intervention. Outcomes assessed after hospital discharge were measured using postal participant questionnaires.

### Outcomes

The primary outcome was the number of days in hospital after surgery [9-13] Secondary outcomes included cost-effectiveness at six months, measured by the Net Benefit statistic, which is calculated using the following equation: ((λ * quality adjusted life year (QALY)) - costs) where λ indicates society's 'willingness to pay' (λ is typically set at £20,000) [24], QALY are calculated using EQ-5D scores and costs included both primary and secondary care costs [25]. Other secondary outcomes, including measures of health status, included changes in health status and quality of life over 6 months after surgery and quality of life at 48 hours, 1, 3 and 6 months after surgery, measured using SF-36 and EQ-5D [25-27]; health care costs including full hospital costs and primary care costs; mortality measured using time-to-event analysis; and the level of major morbidities in hospital using the Post-Operative Morbidity Survey at days 1, 3 and 7 after surgery (POMS) [28] Serious adverse events were assessed by clinical leads defined according to standard definitions, (that is, an untoward occurrence that results in death, is life-threatening, requires hospitalisation or prolongation of existing hospitalisation, results in persistent or significant disability or incapacity, consists of a congenital anomaly or birth defect, or is otherwise considered medically significant by the investigator) [29].

### Sample size

The full power calculation is presented in the trial protocol paper [20]. For the fluid comparison, we aimed to be able to detect a 0.5 SD difference in the primary outcome of number of days in hospital following surgery, with 80% power and 5% significance. This resulted in a target sample size of 128 patients. Data on number of days in hospital were available for all patients in the trial.

### Statistical analysis

The statistical analysis was based on all people randomised, irrespective of subsequent compliance to treatment allocation. Trial analysis was undertaken using standard methods for two-group comparisons for continuous, binary and time-to-event outcomes using intention to treat principles. All statistical analyses were pre-specified in a Statistical Analysis Plan which was agreed upon before the analysis was undertaken. The primary analysis was adjusted for the minimisation variables using analysis of covariance. A significance level of *P *< 0.05 was considered as evidence of statistical significance for the primary outcome and confidence intervals presented. An *a priori *secondary subgroup analysis was investigated through tests for interaction and included patients with high cardiac risk [21]; patients with high-grade functional limitation due to heart failure and type of surgery. Stricter levels of statistical significance (*P *< 0.01) were sought, reflecting the exploratory nature of these subgroup analyses.

### Economic analysis

A within trial economic analysis was conducted from the UK National Health Services' perspective. Discounting was not used since the follow-up was only six months. Benefits in the economic analysis were reported in terms of QALYs estimated from the responses to the EQ-5D questionnaire at five time points - baseline (before surgery), 48 hours, 1 month, 3 months and 6 months after surgery. These responses were converted to utility values using the EQ-5D social tariff, which has been estimated from a representative sample of the UK population [25]. The utility scores obtained at these time points were transformed into QALYs using the area under the curve method by assuming linear extrapolation between subsequent data collection time points.

Patient specific data for the use of NHS resources were retrieved using patient case notes (post-operative inpatient length of stay and outpatient visits) as well as six months' questionnaires to participants (primary care contacts and medicines consumed). Unit costs were retrieved from a number of publicly available sources [30-32]. Intervention cost for base case analysis considered no extra time for pre-operative fluid loading, 30 minutes doctor's time and 42 minutes nurse's time.

Sensitivity analyses were performed for cost and QALYs by alternatively excluding from the analysis participants with 5% and 10% highest and lowest total cost or QALYs. Moreover, the intervention cost was adjusted by adding 12 hours inpatient time in the surgical ward. This scenario would be relevant if normal practice (in the absence of fluid loading) was to conduct surgery on the day of admission. In this situation, patients who receive the fluid intervention would be required to be admitted to hospital earlier to allow time for pre-operative fluid loading to take place. Furthermore, alternative bed-ridden EQ-5D scores were used as baseline utility scores for all participants (that is, -0.402). Finally, missing data were not regarded *a priori *as an issue for the base case analysis. However, this was tested by imputing mean EQ-5D and mean costs for those categories with missing data within each study group and this was further explored using multiple imputation techniques. A base case analysis and sensitivity analyses were performed. For every analysis, differences in mean total cost and mean QALY data were bootstrapped (1,000 repetitions) adjusting for minimisation variables as well as baseline EQ-5D score [33].

### Trial oversight

The Multicentre Research Ethics Committee for Scotland approved the study (Ref 04/MRE10/76). The trial was registered in a public trials registry (registry number ISRCTN32188676). The trial was overseen by a TSC with an independent Chair and an independent DMC reviewed accruing data at regular intervals.

### Role of the funding source

The study sponsor and funding source had no role in the collection, analysis and interpretation of data, or in the writing of the report, or in the decision to submit the paper for publication.

## Results

**Figure 1:**
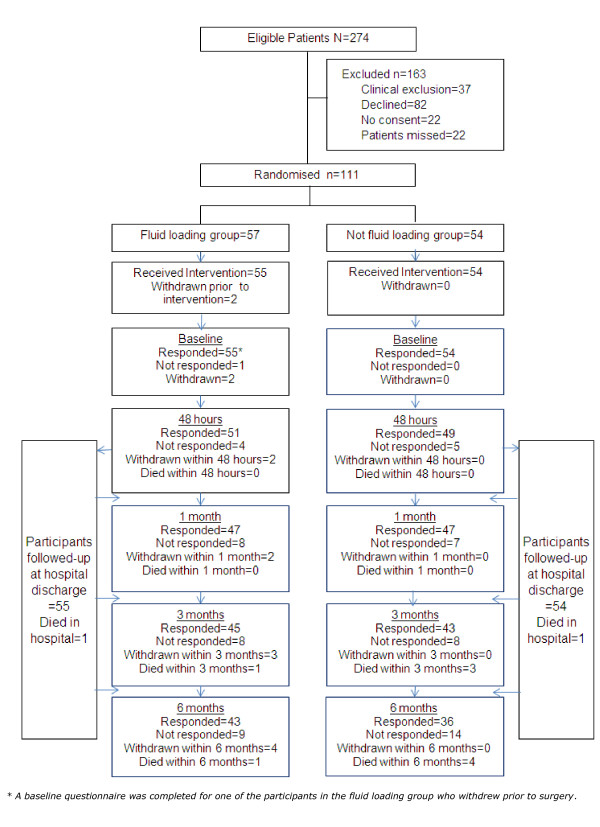
**CONSORT diagram of patient recruitment and retention in the study until final follow-up at six months**. Clinical exclusions: failed to meet inclusion critieria = 13 (emergency surgery (8), inadequate time to deliver intervention (3), not high-risk surgery (2). Presence of exclusion criteria 24 (unspecified clinician concern (8), NYHA grade IV (1), chronic renal failure/creatinine > 300 umol.L^-1 ^(9), unstable angina (1), planned elective admission to ICU (4), surgery scheduled after completion of recruitment (1)).

The study recruited from 1 September 2007 until 4 May 2009. During the study timeframe agreed upon with the funder, a total of 274 patients were deemed eligible for the trial, of whom 111 were recruited. Figure 1 presents the Consolidated Standards on Reporting Trials (CONSORT) diagram and reasons for trial exclusion (163 were excluded for clinical reasons, were missed or did not consent). Of the 111 patients recruited, 57 were randomised to fluid loading and 54 participants to the fluid control group. Further details of the patients' surgical procedures are presented in Additional file 1. In the fluid loading group, one patient withdrew after randomisation but before surgery and a second patient was excluded post-randomisation for clinical reasons (before surgery). All others patients remained in the study until the primary outcome time point. Lost to follow-up for secondary outcomes at six months were four (7%) patients in the fluid loading group and zero (0%) patients in the fluid control group. (These were the two withdrawn before surgery and a further two patients who declined further follow-up at six months). Baseline characteristics are shown in Table 1 and surgical procedures are presented in the Additional file 1.

**Table 1:** Baseline characteristics of the study groups

	Fluid loading (*n *= 57)	Fluid control (*n *= 54)
**Age - median (IQR) years**	69 (64, 78)	73 (64, 80)
**Sex - n (%) male**	41 (72%)	38 (70%)
**Surgical group - n (%)**		
Abdominal with bowel preparation	22 (39%)	26 (48%)
Abdominal without bowel preparation	19 (33%)	17 (31%)
Urological/Gynaecological	8 (14%)	4 (8%)
Vascular	8 (14%)	7 (13%)
**Operative approach - n (%)**		
Open surgery	47 (85%)	38 (70%)
Laparoscopic surgery	6 (11%)	13 (24%)
Not recorded	2 (4%)	3 (6%)
**Analgesia - n (%)**		
Epidural analgesia	39 (71%)	36 (67%)
Patient controlled analgesia (PCA)	15 (27%)	17 (31%)
Combined epidural + PCA	1 (2%)	1 (2%)
**Central venous line monitoring**	42 (76%)	38 (70%)
**Arterial monitoring**	41 (75%)	46 (85%)
**Pre-operative ASA Grade - n (%)**		
2	25 (44%)	22 (41%)
3	30 (53%)	31 (57%)
4	2 (4%)	1 (2%)
**Pre-operative physiological POSSUM score - median (IQR)**	21 (16, 24)	23 (19, 26)
**Revised Cardiac Risk Index^6^- n (%)**		
2	52 (91%)	45 (83%)
> 2	5 (9%)	9 (17%)
**NYHA - n (%)**		
0 to 1	29 (51%)	31 (57%)
2 to 3	27 (47%)	23 (43%)
Missing	1 (2%)	0 (0%)

ASA, American Society of Anaesthesiologists; IQR, Interquartile range; n, number; NYHA, New York Heart Association; POSSUM, Physiologic and Operative Severity Score for the enUmeration of Mortality and Morbidity.

The study interventions and clinical management received in the pre-operative, intra-operative and post-operative periods are presented in Table 2. This table demonstrates the median pre-operative fluid loading volume was 1,875 ml (IQR 1,375 to 2,025) in the fluid loading group compared to 0 (IQR 0 to 0) in the fluid control group. It also demonstrates the median total pre-operative fluid was 1,975 ml (IQR 1,500 to 2,275) in the fluid loading group compared to 0 (IQR 0 to 721) in the fluid control group. The difference between these numbers within the groups was due to pre-operative IV fluid given to replace presumed fluid losses due to oral bowel preparation in line with the protocol. Table 2 also demonstrates that the total IV fluid given in the combined pre and intra-operative periods combined was 4,186 ml (IQR 3,500 to 5,527) in the fluid loading group versus 3,000 ml (IQR 2,500 to 4,050) in the fluid control group. Table 2 also gives details of the time spent in high dependency care or intensive care for each group (treatment received). The mean (SD) preoperative systolic arterial pressure (SAP) was 135 (SD 19) in the fluid loading group and 134 (SD 23) in the controls (*P *= 0.782). Immediately after induction of anaesthesia SAP was 112 (25) in the fluid loading group and 106 (24) in the controls (*P *= 0.386).

**Table 2:** Study interventions and clinical management received in the pre-operative, intra-operative and post-operative periods

	Fluid loading (*n *= 57)		Fluid control (*n *= 54)		
	Median (IQR)	Min, Max	Median (IQR)	Min, Max	*P*-value
Pre-operative fluid load* (ml)	1,875 (1,375, 2,025)	0, 2,950	0 (0, 0)	0, 1,415	< 0.001
**Additional IV fluid load in: bowel preparation**	0		0		
**patients (ml)**	(0, 0)	0, 1,475	(0, 693)	0, 1,500	0.397
**Total pre-operative fluid (ml)**	1,975 (1,500, 2,275)	0, 4,130	0 (0, 721)	0, 1,500	0.000
**Intra-operative fluid (ml)**	2,200 (1,738, 3,500)	970, 7,000	3,000 (2,075, 3,808)	1,000, 6,685	0.078
**Total pre and intra-operative fluid (ml)**	4,186 (3,500, 5,527)	2,000, 9,543	3,000 (2,500, 4,050)	1,000, 7,885	< 0.001
**Total fluid in 24** **hours after surgery (ml)**	3,512 (2,975, 4,356)	1,064, 5,900	3,770 (3,084, 4,434)	1,875, 6,644	0.307
**Total fluid between 24** **and 48 hours after surgery (ml)**	3,000 (2,340, 3,416)	1,320, 6,348	3,219 (2,484, 3,942)	1,650, 4,850	0.235
**Initial post-operative care location (treatment delivered)**	**n (%)**		**n (%)**		
ICU care	14 (25%)		7 (13%)		
HDU care	35 (64%)		46 (85%)		
Ward care	6 (11%)		1 (2%)		
	**Median (IQR)**	**Min, Max**	**Median (IQR)**	**Min, Max**	***P*-value**
**Total time in ICU after** **surgery (hrs)**	0 (0, 7.2)	0, 701.2	0 (0, 0)	0, 331.0	0.327
**Total time in HDU after** **surgery (hrs)**	71.5 (44.0, 120.2)	0, 580.3	92.9 (67.4, 148.2)	0, 617.4	0.028
**Total time in Ward after surgery (hrs)**	122.5 (89.3, 216.5)	22.3, 1198.5	164.5 (90.9, 303.8)	5.0, 3080.0	0.204

Hrs, hours; IQR, Interquartile range; ml: millilitres.

*Two participants in the fluid loading group were treated as part of the trial but received no pre-operative fluid loading and four participants in the fluid control group received pre-operative fluid loading.

There were no serious adverse events leading to persistent or significant disability or incapacity in either group. Serious adverse events prolonging hospital stay were seen in 6 (11%) patients in the fluid loading group and 14 (26%) patients in the fluid control group (*P *= 0.048). Of these, six were life threatening with two (4%) patients in the fluid loading group and three (6%) patients in the fluid control group (*P *= 0.673). The adverse events were cardiac (2 in the fluid group, 1 in the control group), arrhythmias (1 vs 0), gastrointestinal (2 vs 5), infectious (1 vs 5), other (0 vs 1). More details are presented in Additional file 2.

Table 3 demonstrates the primary trial outcome of days in hospital after surgery. This table demonstrates a mean hospital stay of 12.2 (SD 11.5) days in the fluid loading group compared to 17.4 (SD 20.0) in the fluid control group with an adjusted mean difference of 5.5 days (median 2.2 days; bootstrapped 95% CI -0.44 to 11.44; *P *= 0.07). Similar effect sizes are seen for hospital admission until hospital discharge (mean difference: 5.99; CI -0.19 to 12.18) and for days from randomisation until hospital discharge (mean difference: 9.03; CI 0.96 to 17.11).

**Table 3:** The primary trial outcome of days in hospital after surgery

	Fluid loading (*n *= 55)	Fluid control (*n *= 54)	Effect size	Bootstrap 95% CI	*P*-value
**Days in hospital after surgery**					
mean (SD)	12.2 (11.5)	17.4 (20.0)	5.50	-0.44, 11.44	0.070
median (IQR)	8.8 (6.9,13.0)	11.0 (7.8, 18.9)	2.2		

CI, confidence interval; IQR, interquartile range; SD, standard deviation.

The effect size is the difference in means adjusted for minimisation covariates.

Table 4 demonstrates secondary outcomes including post-operative morbidity measured using the POMS score [28] at one, three and seven days and operative morbidity measured using the operative POSSUM score [23] and crude mortality at hospital discharge, three months and six months. No patients required mechanical ventilation after surgery, with all patients being extubated in the operating room immediately after surgery.

**Table 4:** Trial secondary outcomes

	Fluid loading	Fluid control	**Odds ratio**†	95% CI	*P*-value
**Operative POSSUM score - median (IQR)**	12 (9,16)	12 (9,16)	na*	na	na
**POMS outcomes**					
Day 1 POMS morbidity	55 (100%)	53 (98%)	na**	na	na
- Day 3 POMS morbidity	52 (94%)	50 (93%)	1.604	0.277 to 9.279	0.598
- Day 7 POMS morbidity***	23 (51%)	31 (67%)	0.515***	0.200 to 1.326	0.169
**Hospital mortality**	1 (2%)	1 (2%)	0.981	0.012 to 78.476	1.000
**3-month mortality**	1 (2%)	3 (6%)	0.315	0.006 to 4.106	0.994
**6-month mortality**	1 (2%)	4 (8%)	0.231	0.005 to 2.471	0.995

CI,: confidence interval; IQR, Interquartile range; POMS, post-operative morbidity score; POSSUM, Physiologic and Operative Severity Score for the enUmeration of Mortality and Morbidity.

* statistical significance not tested due to small number of events. ** 100% incidence in fluid group makes testing inappropriate. *** 19 participants (10 in fluid group and 9 in non-fluid group) were discharged less six days after surgery.

†Odds ratios adjusted for minimisation covariates. Post-operative morbidity measured using POMS^28 ^on days 1, 3 and 7 and operative complexity measured using operative POSSUM score^23 ^and crude mortality at hospital discharge, 3 months and 6 months.

Table 5 shows cost effectiveness results for the base case analysis. Pre-operative fluid loading on average costs less (£10,373) than standard care (£11,739) with an adjusted mean difference of -£2,047 (bootstrapped 95% CI: -£6,947 to £2,854; *P *= 0.413). Moreover, pre-operative fluid loading on average is also more effective (QALY = 0.3527) than no fluid loading (QALY = 0.3175) with an adjusted mean difference of 0.0431 (bootstrapped 95% CI: -0.0171 to 0.1033; *P *= 0.161); this difference being equivalent to about 7.87 days over a 6-month trial follow-up period. Table 5 illustrates a high probability that the intervention is dominant compared with a no fluid loading approach. The CEAC shows that there is a high probability that the intervention will be cost-effective at all the threshold values of willingness to pay for a QALY presented (table 5).

**Table 5:** The base case economic evaluation results

Base case ICER calculations						Threshold analysis*: probability of cost-effective at alternative values of willingness to pay for a QALY (%)			
	**Mean total cost (£)**	**Incremental cost (£)**	**Mean QALY**	**Incremental QALY**	**ICER (£/QALY)**	**£10,** **000**	**£20,** **000**	**£30,** **000**	**£50,** **000**

**Fluid loading**	10,373		0.3527			84.4	86.5	89.4	92.0
**Fluid control**	11,739	-1,366	0.3175	0.0352	Dominated	15.6	13.5	10.6	8.0
Adjusted differences (95% CI; *P-*value)*		-2,047 (-6,947 to 2,854; *P *= 0.413)		0.0431 (-0.0171 to 0.1033; *P *= 0.161)					

CI, confidence interval; ICER, incremental cost-effectiveness ratio; QALY, quality adjusted life year.

* Adjusted for minimisation variables and baseline EQ-5D score and based on 1,000 bootstrap iterations.

Sensitivity analyses were conducted on both the cost and QALY calculations (data not shown). The fluid intervention remains dominant (less costly and more effective) for all but one of the sensitivity analyses considered. In this analysis, we trimmed the 5% most costly data from the no fluid arm of the trial. In this case, the intervention was more costly and more effective, reflected in an incremental cost-effectiveness ratio (ICER) of £24,810 per QALY gained. This analysis represents a worst case scenario, in which fluid loading is still cost effective as the ICER is less than the £30,000 per QALY threshold commonly recommended by the National Institute of Health and Clinical Excellence (NICE). These would, however, reflect clinically important patients, and their exclusion from the analysis may not be a correct representation of the population being studied. Nonetheless, in this one analysis fluid loading was associated with an ICER of less than £30,000, a value commonly adopted by NICE for technology appraisal. Conclusions remained unaltered for the "day of surgery admission" sensitivity analysis. This further highlights the high likelihood of cost-effectiveness of the interventions across a variety of assumptions.

Subgroup analysis of the effects of cardiac risk, heart failure grade and type of surgery showed no evidence of interaction with the main effect of the intervention (data not shown).

## Discussion

The study demonstrated a non-significant trend towards a reduction of hospital length of stay with a mean difference of 5.50 (-0.44, 11.44) days (median 2.20 days). This direction of effect was also observed when considering total duration of hospital stay and days from study randomisation until hospital discharge. Observed differences towards improvements in secondary outcome measures also support the proposition that fluid loading is likely to be beneficial by showing that pre-operative fluid loading tended towards reduced serious adverse events that prolonged hospital stay and resulted in a non-significant reduction in post-operative morbidity (at seven days). The finding of the economic analysis was that fluid loading results in, on average, lower costs and greater benefits than no fluid loading. Cost savings associated with the intervention were mainly driven by longer post-operative inpatient length of stay in the fluid control group (data not shown). Based on the balance of probabilities; there is a high probability that fluid loading is cost-effective compared to fluid control.

### The fluid intervention

The fluid loading group received a median of 1,875 ml of Ringer's solution in the pre-operative period compared to a median of 0 ml in the control; this correlates well with the 25 ml/kg protocolised target pre-operative fluid load. This fluid was delivered over a six-hour period before surgery. With clinical practice changing towards day of surgery admission in many countries, future studies in this area may find it difficult to deliver this intervention and may choose to consider testing whether shorter periods of fluid administration are appropriate; for instance, delivery of the fluid intervention in a two-hour period rather than six. It is important to identify that our sensitivity analysis suggests that the intervention is still highly likely to be cost-effective even if patients, who would normally be admitted on the day of surgery, had to be admitted to hospital up to 12 hours earlier (that is, the night before surgery) to receive the intervention.

With regard to the choice of fluid used, we chose Ringer's solution due to our desire to select a crystalloid and use a balanced solution. We believe this was the appropriate choice and would be unlikely to change this selection in a future study. In this study we specifically documented cardiac adverse events as being potentially important morbidities related to the fluid intervention but there was no evidence of increased cardiac adverse events associated with fluid loading and, indeed, there was a reduction in overall adverse events in the fluid group. We do not have direct data to explain why there were fewer gastro-intestinal and infectious adverse events in the fluid group but this may be explained by an improvement in cardiac output and oxygen delivery that would be expected in patients having fluid loading [3-7]. This may improve tissue (including gut blood flow) and reduce anastomotic breakdown and reduce tissue infection. Some centres use cardiac output guided therapy to guide fluid therapy in such patients and it will be important to test this fluid intervention combined with, or compared to such peri-operative strategies. Despite the lack of clinical evidence of fluid related complications in this study the use of more invasive monitoring in future studies may allow a more full investigation of this issue.

### What this study adds

We hypothesised that simple pre-operative fluid loading would represent a straightforward and cost-effective way to shorten stay and improve outcome after major high risk surgery. We designed the intervention to be as simple and pragmatic as possible by delivering a fixed "dose" (25 ml/kg) of intravenous Ringer's lactate solution in the six hours before surgery in the ward environment without the requirement to site, monitor or target the complex cardiovascular targets that cardiac output devices allow. This intervention was designed to be easily protocolized for clinical practice and be delivered by non-medical staff to further enhance its utility.

We have demonstrated a trend towards a reduction in hospital length of stay after surgery and that the fluid intervention is likely to be cost-effective. This is supported by concomitant observed reductions across both the high dependency and ward length of stay, both of which are reduced in the fluid loading group. We also demonstrated that fluid loading was associated with short term reductions in adverse events as well as improvements in longer term (six-month) outcomes, such as the effectiveness of care, reductions in health care costs and improved cost-effectiveness as well. All these secondary outcomes show effects in the direction of benefit for the intervention and help us understand the contributing factors that are associated with improvements in outcome.

The mechanisms by which this fluid intervention appears to reduce hospital length of stay are not entirely clear from the results of the study. We know that the fluid loading group received the pre-operative fluid intervention as per protocol and received a significantly greater amount of fluid before surgery commenced. This group was also found to have received more fluid by the end of surgery than the fluid control group. Therefore, these patients received more fluid, earlier, than controls and much of this fluid was administered before the surgical insult. Pre-operative optimisation studies often demonstrate an increase in cardiac index and oxygen delivery with fluid loading alone, which is further augmented by inotropic support to achieve supranormal oxygen delivery targets [3-7]. We would hypothesis that pre-operative fluid loading improves cardiac output and oxygen delivery, but to levels below "supranormal" levels [3,5,34], and this is associated with improved organ perfusion and function [3,5,34], fewer surgical complications [35] and fewer adverse events, lower post-operative morbidity, and these factors contribute to the shorter length of stay in hospital after surgery. The magnitude of reduction in hospital length of stay is similar to that seen in other optimisation studies [5,9-11]. It should be noted that this study, and the pre-optimisation literature, appears to contradict the evidence base for intra-operative fluid restriction, which has appeared more recently [15-19]. However, the apparent discrepancy between these two bodies of evidence may be less difficult to reconcile than it appears. There are seven randomised studies in the literature on fluid restriction and of these only three show benefit [36]. Evidence from the first study of fluid restriction strategies suggests that restricting day of surgery fluid intake from approximately 6,200 ml (of which 5,388 ml were IV) to approximately 3,700 ml (of which 2,740 ml were IV) may be beneficial in terms of complications [15]. Whereas, the current study, and many of the studies cited in the pre-operative optimisation literature, utilise peri-operative fluid loads of 3,000 to 4,000 ml [5]. In studies of fluid restriction, a range of a "liberal intra-operative fluid regimens" from 2,750 to 5,388 ml compared with 998 to 2,740 ml for the "restrictive fluid regimen"[36]. This may suggest that "restrictive fluid regimens" may not actually differ that significantly from optimization strategies in terms of fluid volume. The difference that may explain these two apparently contradictory strategies may relate to either the timing of the fluid administration (early preoperative fluid loading being beneficial and late post-operative fluid overloading being harmful) or related to the achievement of "supranormal" targets. In the Noblett study, which utilised an intra-operative fluid optimisation regime, a significant majority of the fluid administration occurred in the first 40 minutes of surgery [9]. Therefore, there could be an argument that we should target early (pre- and early intra-operative) fluid loading/optimisation and then move to target late (end of surgery) active fluid restriction to avoid post-operative fluid overload and late complications. These bodies of evidence may, therefore, be complementary and not contradictory [37].

If this reduction in hospital length of stay can be replicated in a larger study then this finding will have a major impact on service delivery and resource allocation. From a patient's perspective this could allow them to get back to their home environment earlier. The intervention may also lead to less adverse events and other acute morbidities, which would clearly have direct patient benefit. From a clinical practice perspective, this intervention would be simple, inexpensive and is likely to be cost-effective as well as being relatively easy to implement in acute hospitals. There would be little educational support required for implementation as the knowledge and skills are already present in surgical teams in all acute hospitals. The intervention could be easily protocolized and nurse-led to allow reliable and reproducible delivery in practice. This reduction in length of stay would lead to significant direct savings in clinical budgets and allow the re-allocation of these resources. The simplicity and low cost of this intervention may make it more attractive than other optimisation strategies [3-14].

### Strengths and weaknesses

One of the main strengths of this trial is the importance and simplicity of the clinical research question. We believe this is the first randomised controlled trial of fluid loading in high risk major surgery. The multi-centre nature of the study adds to the generalizability of the study results. The simplicity and low cost of the fluid intervention are key factors making this intervention comparatively simple to implement into surgical practice internationally. An integral part of the study was a prospective cost-effectiveness analysis that is unique in this clinical field and still uncommon in randomised clinical trials in acute care. The economic evaluation has been conducted using the best available methods, including an extensive and detailed costing approach. This analysis suggests that the fluid loading intervention is highly likely to be cost-effective, adding greatly to the importance and impact of the study. However, this study was not powered to detect a difference in cost-effectiveness between groups. Therefore, it is perhaps not surprising that the evidence on cost-effectiveness falls short of conventional levels of statistical significance. Consideration has, therefore, been given to the balance of probabilities when drawing conclusions about cost-effectiveness. There was some minor imbalance between groups with regard to baseline characteristics, such as age and number of patients undergoing abdominal surgery with bowel preparation. A slightly higher number of patients in the fluid intervention group received ICU care in the early post-operative period and this could be argued to introduce a bias in favour of the intervention group by improving the care delivered to this group. This difference is believed to have occurred by chance and not be driven by clinical issues, including no increase in the requirement for post-operative ventilation and no major differences in surgery performed. The definition of high risk status varies between studies and we chose to use one of the most widely used RCRI [21]. Despite the precise application of this score we still recruited patients with American Society of Anesthesiologists (ASA) grades of 2 and, therefore, apparent low to moderate risk patients. This does not represent a misclassification of patients but does mean the study group is moderate to high risk by some clinical risk criteria. We believe the external generalizability of this study is high. Such high risk patients undergo major surgery in all countries and they have a significant morbidity and mortality. This intervention is applicable and implementable in all developed countries but many countries have day of surgery admission. Since the loading dose of fluid is relatively modest, it does not seem unreasonable to think this intervention could be delivered in the two to three hours before surgery for patients admitted on the day of surgery. Further, our sensitivity analysis suggests that it would still be highly likely to be cost-effective to admit such patients up to 12 hours before surgery (the night before) to receive their fluid intervention. Our median total length of stay for this surgery seems to be broadly in line with UK averages and other studies [14,38], although above those lengths of stays seen in "fast track surgery studies" [15,18,36]. A further significant weakness was that our final recruitment total was short of our desired sample size (111 instead of 128), which meant that we were slightly underpowered to detect our *a priori *proposed minimally important difference of 0.5 SD (111 patients gave 80% power to detect 0.54 SD change rather than 0.5 SD).

## Conclusions

We conclude that, when applied to high-risk major surgery patients, pre-operative intravenous fluid loading with 25 ml/kg Ringer's lactate solution may lead to a clinically important reduction in hospital length of stay after surgery. Further, pre-operative fluid loading is likely to be cost-effective. Further confirmatory work is required to determine whether this effect is important and reproducible. We suggest that this be conducted in the form of a multi-centre, randomised, controlled trial of pre-operative fluid loading in high-risk major surgery with the power to test the effects of the intervention on hospital morbidity and mortality, and hospital length of stay, as well as cost-effectiveness of care. If these results are confirmed, then this simple intervention can allow more effective and less expensive delivery of surgical services.

## Key messages

• Fluid optimisation around the time of major surgery is a controversial issue with evidence supporting pre-optimisation and supranormalisation and also favouring fluid restriction.

• One of the key elements of pre-optimisation is fluid loading before surgery. There is little evidence to guide practice on the role of simple fluid loading alone in preparation for surgery.

• We performed an RCT of fluid loading before major surgery and have demonstrated that there is a potential for benefit in terms of hospital stay and cost-effectiveness for this therapy.

• Further studies are required to corroborate these results.

## Abbreviations

λ: society's 'willingness to pay'; ASA: American Society of Anesthesiologists; CI: confidence interval; DMC: Data Monitoring Committee; EQ-5D: EuroQOL 5D quality of life metric; ICER: incremental cost-effectiveness ratio; ICU: intensive care unit; IQR: interquartile range; kg: kilograms; ml: millilitres; NICE: National Institute of Health and Clinical Excellence; POMS: Post-Operative Morbidity score; POSSUM: Pre-Operative and Operative Physiological and Operative Severity Score for Enumeration of Mortality and Morbidity; QALY: quality adjusted life year; RCRI: Revised Cardiac Risk Index; RCT: randomised controlled trial; SAP: systolic arterial pressure; SD: standard deviation; SF-36: short form 36; TSC: Trial Safety Committee.

## Competing interests

The authors declare that they have no competing interests.

## Authors' contributions

BHC, MKC, SAS, DR and DA have participated fully in the design of this study, the collection and analysis of data, and in the writing of the paper. AE and DB have participated fully in analysis of the data and in the writing of the paper. RH, JN, JK, JB, JC and AG have participated fully in the design of this study, the analysis of the data, and in the writing of this paper. SCC and JA have participated fully in the collection of data, analysis of the data, and in the writing of the paper. All authors have seen and approved the final version of the paper.

## Supplementary Material

Additional file 1 **Surgical procedure for trial participants by minimisation group**. Click here for file

Additional file 2 **The effect of adverse events and on length of stay according to surgical procedure**. Click here for file

Additional file 3 **FOCCUS collaborators' group**. Click here for file
