# Supplementary material for: A pragmatic multi-centre randomised controlled trial of fluid loading in high-risk surgical patients undergoing major elective surgery - the FOCCUS study
Source: Crit Care. 2011 Dec 16;15(6):R296. doi: 10.1186/cc10592 (PMC3388651; doi:10.1186/cc10592)
Supplement: Additional file 1 — Surgical procedure for trial participants by minimisation group. [file cc10592-S1.DOC]

**Appendix 1- Surgical procedure for trial participants by minimisation group**

|  | **Abdominal with bowel preparation** | | | **Abdominal without bowel preparation** | | | **Urological / Gynaecological** | | | **Vascular** | | | **All surgery** | | |
| --- | --- | --- | --- | --- | --- | --- | --- | --- | --- | --- | --- | --- | --- | --- | --- |
|  |  |  |  |  |  |  |  |  |  |  |  |  |  |  |  |
|  | Fluid loading | Fluid control | All | Fluid loading | Fluid control | All | Fluid loading | Fluid control | All | Fluid loading | Fluid control | All | Fluid loading | Fluid control | All |
| Abdominal Hysterectomy | 0 | 0 | 0 | 0 | 0 | 0 | 0 | 1 | 1 | 0 | 0 | 0 | 0 | 1 | 1 |
| Abdominal Periexcision | 0 | 0 | 0 | 3 | 0 | 3 | 0 | 0 | 0 | 0 | 0 | 0 | 3 | 0 | 3 |
| Anterior Resection | 2 | 7 | 9 | 10 | 5 | 15 | 0 | 0 | 0 | 0 | 0 | 0 | 12 | 12 | 24 |
| Aorta Bifemoral Bypass Graft | 0 | 0 | 0 | 0 | 0 | 0 | 0 | 0 | 0 | 1 | 1 | 2 | 1 | 1 | 2 |
| Aorta exploration | 0 | 0 | 0 | 0 | 0 | 0 | 0 | 0 | 0 | 1 | 0 | 1 | 1 | 0 | 1 |
| Aortic Aneurysm | 0 | 0 | 0 | 0 | 0 | 0 | 0 | 0 | 0 | 5 | 5 | 10 | 5 | 5 | 10 |
| Aortic Aneurysm / Anterior Resection | 0 | 0 | 0 | 0 | 0 | 0 | 0 | 0 | 0 | 0 | 1 | 1 | 0 | 1 | 1 |
| Aorto Biprofunda Bypass Graft | 0 | 0 | 0 | 0 | 0 | 0 | 0 | 0 | 0 | 1 | 0 | 1 | 1 | 0 | 1 |
| Completion Colectomy | 1 | 0 | 1 | 0 | 0 | 0 | 0 | 0 | 0 | 0 | 0 | 0 | 1 | 0 | 1 |
| Cystectomy / Anterior Resection | 0 | 0 | 0 | 0 | 0 | 0 | 1 | 0 | 1 | 0 | 0 | 0 | 1 | 0 | 1 |
| Cystectomy / Ileal Conduit | 0 | 0 | 0 | 0 | 0 | 0 | 1 | 1 | 2 | 0 | 0 | 0 | 1 | 1 | 2 |
| Frey's procedure / Splenectomy | 1 | 0 | 1 | 0 | 0 | 0 | 0 | 0 | 0 | 0 | 0 | 0 | 1 | 0 | 1 |
| Gastrectomy | 2 | 0 | 2 | 0 | 0 | 0 | 0 | 0 | 0 | 0 | 0 | 0 | 2 | 0 | 2 |
| Gastrojejunostomy | 0 | 1 | 1 | 0 | 0 | 0 | 0 | 0 | 0 | 0 | 0 | 0 | 0 | 1 | 1 |
| Hartmann's Reversal | 0 | 0 | 0 | 0 | 1 | 1 | 0 | 0 | 0 | 0 | 0 | 0 | 0 | 1 | 1 |
| Hemicolectomy | 12 | 7 | 19 | 3 | 5 | 8 | 0 | 0 | 0 | 0 | 0 | 0 | 15 | 12 | 27 |
| Ileo-sigmoid anastamosis | 0 | 1 | 1 | 0 | 0 | 0 | 0 | 0 | 0 | 0 | 0 | 0 | 0 | 1 | 1 |
| Laparotomy | 1 | 5 | 6 | 0 | 0 | 0 | 0 | 0 | 0 | 0 | 0 | 0 | 1 | 5 | 6 |
| Nephrectomy or Nephro-uretherectomy | 0 | 0 | 0 | 0 | 0 | 0 | 4 | 1 | 5 | 0 | 0 | 0 | 4 | 1 | 5 |
| Oesophagectomy | 1 | 0 | 1 | 0 | 0 | 0 | 0 | 1 | 1 | 0 | 0 | 0 | 1 | 1 | 2 |
| Pancreaticoduodenectomy | 0 | 2 | 2 | 0 | 0 | 0 | 0 | 0 | 0 | 0 | 0 | 0 | 0 | 2 | 2 |
| Radical prostatectomy | 0 | 0 | 0 | 0 | 0 | 0 | 2 | 0 | 2 | 0 | 0 | 0 | 2 | 0 | 2 |
| Retroperitoneal | 0 | 0 | 0 | 1 | 0 | 1 | 0 | 0 | 0 | 0 | 0 | 0 | 1 | 0 | 1 |
| Sigmoid Colectomy | 0 | 3 | 3 | 2 | 4 | 6 | 0 | 0 | 0 | 0 | 0 | 0 | 2 | 7 | 9 |
| Sigmoid Colectomy / Nephrectomy | 0 | 0 | 0 | 0 | 1 | 1 | 0 | 0 | 0 | 0 | 0 | 0 | 0 | 1 | 1 |
| Small bowel resection | 0 | 0 | 0 | 0 | 1 | 1 | 0 | 0 | 0 | 0 | 0 | 0 | 0 | 1 | 1 |
| No surgery performed | 2 | 0 | 0 | 0 | 0 | 0 | 0 | 0 | 0 | 0 | 0 | 0 | 0 | 0 | 0 |
|  | **22** | **26** | **48** | **19** | **17** | **36** | **8** | **4** | **12** | **8** | **7** | **15** | **57** | **54** | **111** |
