# Supplementary material for: A pragmatic multi-centre randomised controlled trial of fluid loading in high-risk surgical patients undergoing major elective surgery - the FOCCUS study
Source: Crit Care. 2011 Dec 16;15(6):R296. doi: 10.1186/cc10592 (PMC3388651; doi:10.1186/cc10592)
Supplement: Additional file 2 — The effect of adverse events and on length of stay according to surgical procedure. [file cc10592-S2.DOC]

**Appendix 2-** The effect of adverse events and on length of stay according to surgical procedure.

|  | Number of operations | Study group | Mean LOS (days) | Number of patients with SAE | Total number of  SAEs | Mean LOS - days  (patients with SAE) |
| --- | --- | --- | --- | --- | --- | --- |
| Hemicolectomy | 27 | 15  fluid | 14.8 | 1 | 5  (Cardiac 0, Arrhythmia 1, GI 2, infectious 2, Other 0) | 72.5 |
|  |  | 12  non-fluid | 11.1 | 2 | 2  (Cardiac 0, Arrhythmia 0, GI 1, infectious 1, Other 0) | 18.5 |
| Anterior resection | 24 | 12  fluid | 11.4 | 4 | 4  (Cardiac 2, Arrhythmia 0, GI 1, infectious 1, Other 0) | 15.0 |
|  |  | 12  non-fluid | 18.6 | 5 | 8  (Cardiac 1, Arrhythmia 0, GI 2, infectious 3, Other 2) | 27.5 |
| Abdominal aortic aneurysm | 10 | 5  fluid | 17.7 | 1 | 1  (Cardiac 0, Arrhythmia 0, GI 0, infectious 1, Other 0) | 9.1 |
|  |  | 5  non-fluid | 8.4 | 0 | 0  (Cardiac 0, Arrhythmia 0, GI 0, infectious 0, Other 0) | n/a |
| Laparotomy | 6 | 1  fluid | 9.9 | 0 | 0  (Cardiac 0, Arrhythmia 0, GI 0, infectious 0, Other 0) | n/a |
|  |  | 5  non-fluid | 48.8 | 2 | 2  (Cardiac 0, Arrhythmia 0, GI 1, infectious 1, Other 0) | 94.6 |
| Sigmoid colectomy | 9 | 2  fluid | 7.8 | 0 | 0  (Cardiac 0, Arrhythmia 0, GI 0 infectious 0, Other 0) | n/a |
|  |  | 7  non-fluid | 15.3 | 2 | 2  (Cardiac 0, Arrhythmia 1, GI 0, infectious 0, Other 1) | 19.5 |
