# Supplementary material for: A pragmatic multi-centre randomised controlled trial of fluid loading in high-risk surgical patients undergoing major elective surgery - the FOCCUS study
Source: Crit Care. 2011 Dec 16;15(6):R296. doi: 10.1186/cc10592 (PMC3388651; doi:10.1186/cc10592)
Supplement: Additional file 3 — FOCCUS collaborators' group. [file cc10592-S3.DOC]

**Appendix 3- *FOCCUS collaborators group***

Brian H Cuthbertson^1,2^, Marion K Campbell^2^, Stephen A Stott^4^, Andrew Elders^2^, Rodolfo Hernández^2,3^, Dwayne Boyers^3^, John Norrie^5^, John Kinsella^6^, Julie Brittenden^7^, Jonathan Cook^2^, Daniela Rae^2^, Seonaidh C Cotton^2^, David Alcorn^8^, Jennifer Addison^2^, Adrian Grant^9^, Alison MacDonald^2^, Gladys MacPherson^2^, Graeme MacLennan^2^, Luke Vale^3^, Kirsty Shepherd^6^, Anne Marie McColl^8^, Michael Serpell^10^.

1. Department of Critical Care Medicine, Sunnybrook Health Sciences Centre, 2075 Bayview Avenue, Room D132, Toronto, Ontario, Canada, M4N 3M5.
2. Health Services Research Unit, Health Sciences Building, University of Aberdeen, Ashgrove Road, Foresterhill, Aberdeen, Scotland, UK, AB25 2ZD.
3. Health Economics Research Unit, Polwarth Building, University of Aberdeen, Foresterhill, Aberdeen, Scotland, UK, AB25 2ZD.
4. Intensive Care Unit, Aberdeen Royal Infirmary, Westburn Road, Aberdeen, Scotland, UK, AB25 2ZN.
5. Robertson Centre for Biostatistics, Boyd Orr Building, University of Glasgow, Glasgow, UK, G12 8QQ.
6. Section of Anaesthesia Pain and Critical Care, University of Glasgow, 4th Floor, Walton Building, Glasgow Royal Infirmary, 91 Wishart Street, Glasgow, UK, G31 2HT.
7. Department of Surgery, University of Aberdeen, Polwarth Building, Foresterhill Aberdeen, Scotland, UK, AB25 2ZD.
8. Royal Alexandra Hospital, Corsebar Road, Paisley, Strathclyde, Scotland, UK, PA2 9PN.
9. Institute of Applied Health Sciences, Health Sciences Building, University of Aberdeen, King’s College, Foresterhill, Aberdeen, Scotland, UK, AB25 2ZD.
10. Western Infirmary, Dumbarton Road, Glasgow, UK, G11 6NT.

***Independent chair of the steering group –*** Dr. Duncan Young, University of Oxford, UK.

***Data monitoring committee –*** Prof. Julian Bion (Chair), University of Birmingham, Birmingham, UK; Prof. Diana Elbourne, London School of Hygiene and Tropical Medicine, London, UK; Dr. Sue Todd, University of Reading, Reading, UK.
